# Re: Arcuri and Americo “Treatment of relapsed/refractory multiple myeloma in the bortezomib and lenalidomide era: a systematic review and network meta-analysis”

**DOI:** 10.1007/s00277-022-04792-0

**Published:** 2022-02-18

**Authors:** Faith E. Davies, Eleanor Saunders, François Bourhis, Patricia Guyot

**Affiliations:** 1 Perlmutter Cancer Center, NYU Langone Health, New York City, NY USA; 2 Sanofi, Thames Valley Park Drive, Reading, Berkshire UK; 3 Sanofi, Chilly-Mazarin, France

Dear Editor,

We read with interest the article “Treatment of relapsed/refractory multiple myeloma in the bortezomib and lenalidomide era: a systematic review and network meta-analysis” (*Ann Hematol* 100, 725–734 [2021]). Such an article is important for decision-making to inform clinicians of the most effective and safe treatments in a complex setting such as relapsed refractory multiple myeloma (MM). However, all such studies should be conducted in line with recognized guidance [1, 2], and caution is warranted in the interpretation of this study as there are a number of serious methodological and data concerns that limit the validity of the conclusions.

The systematic literature review that was conducted to identify phase III studies was originally restricted to include only lenalidomide or bortezomib in the control arm. Fifteen such studies were identified. However, this protocol was violated when “two studies with pomalidomide and one with carfilzomib in the control arm were also included.”

Furthermore, the principles which underpin the network meta-analysis (NMA) methodology are violated on multiple levels:

- Trials evaluating different populations without any adjustment for treatment effect modifiers are compared.
- Studies were conducted over at least a 15-year period, during which drug availability and standard of care treatment regimens varied considerably.
- Different backbone therapies are “considered equivalent therapies” and combined as a single “control” group.
- Severe adverse events (SAE) are assumed to be comparable with grade III/IV events.

It is well documented that patient characteristics (i.e., age, performance status, cytogenetic risk), number of prior treatment lines, type of therapies received, and refractoriness to treatment options may act as prognostic factors and treatment effect modifiers. However, study inclusion criteria vary from no refractory patients (DOXIL-MMY-3001) to all patients were refractory (ICARIA), and the median number of prior lines of therapy ranged from 1 to 3 (see Table 1).

**Table 1:** Overview of the studies included in the NMA by Arcuri and Americo (2021)

Study	Intervention (*n*)	Control (*n*)	Patient population	Median number of prior treatment lines	Bortezomib exposed	Bortezomib refractory	Lenalidomide exposed	Lenalidomide refractory	Refractory to lenalidomide and bortezomib	Adverse events source in NMA
VANTAGE 088 [3]	VorV (*n* = 317)	V (*n* = 320)	1–3 prior regimens	2 prior regimens	+	−	+	−	−	SAE
POLLUX [4]	DRd (*n* = 286)	Rd (*n* = 283)	1 +	1 (1–11)	+ + + ^*^	+ ^*^	+ + ^†^	+ ^‡^	−	SAE
ENDEAVOR [5]	Kd (*n* = 464)	Vd (*n* = 465)	1 +	2 (1–2)	+ +	−	+ +	−	−	SAE
TOURMALINE-MM1 [6]	NRd (*n* = 360)	Rd (*n* = 362)	1–3	1 prior: 62%	+ + +	+	+	−	−	SAE
TOURMALINE MM1-China [7]	NRd (*n* = 57)	Rd (*n* = 58)	1–3	1 prior: 44%	+ +	+	+	−	−	SAE
NCT00813150 [8]	CyVd (*n* = 46)	Vd (*n* = 47)	1 +	1 prior: 57%	NR	NR	NR	NR	NR	SAE
ELOQUENT-2 [9]	ERd (*n* = 321)	Rd (*n* = 325)	1–3	2 (1–4)	+ + +	+	+	−	−	SAE
KEYNOTE-183 [10]	PembroPd (*n* = 125)	Pd (*n* = 124)	2 + including IMiD and PIs	3 (1–3)	+ + +	NR	+ + +	+ + +	+ + ^§^	SAE
DOXIL-MMY-3001 [11]	PEG-Dox (*n* = 324)	V (*n* = 322)	1 +	66% received 2 + therapies	−	−	−	−	−	SAE
CASTOR [12]	DVd (*n* = 251)	Vd (*n* = 247)	1 +	2 (1–9)	+ + ^*^	−	+ + ^†^	+	−	Grade III/IV
OPTIMISMM [13]	PVd (*n* = 281)	Vd (*n* = 278)	1–3 and R-refractory	NR (1–3)	+ + +	+	+ + +	+ + +	NR	SAE
PANORAMA-1 [14]	PanVd (*n* = 387)	Vd (*n* = 381)	1–3 treatments	1 (1–3)	+ +	+	+	NR	NR	SAE
ASPIRE [15]	KRd (*n* = 396)	Rd (*n* = 396)	1–3	2 (1–3)	+ +	−	+	−	−	SAE
BELLINI [16]	VenVd (*n* = 194)	Vd (*n* = 97)	1–3	NR^¶^	+ + ^*^	NR	+ +	+	NR	SAE
GMMG ReLApsE [17]	ASCT-Rd (*n* = 139)	Rd (*n* = 138)	1–3	1 prior: 94%	+ + +	−	+	−	−	SAE
BOSTON [18]	SVd (*n* = 195)	Vd (*n* = 207)	1–3 prior regimens	2 (1–2)	+ + +	−	+ +	−	−	SAE but stated as NR
CANDOR [19]	DKd (*n* = 312)	Kd (*n* = 154)	1–3 prior therapies	2 (1–2)	+ + +	+	+ +	+	NR	SAE
ICARIA-MM [20]	IsaPd (*n* = 154)	Pd (*n* = 153)	2 + and have not responded to R or a PI	3 (2–4)	+ + +	+ + +	+ + +	+ + +	+ + +	Grade III/IV

+ 1–33%; +  + 34–66%; +  +  + 67–100%

− 0%

^*^Based on prior exposure to a proteasome inhibitor.

^†^Based on prior exposure to an immunomodulatory drug.

^‡^Trial excluded lenalidomide refractory patients however some patients appear to have been enrolled.

^§^Patients were considered refractory if two (double: lenalidomide and bortezomib), three (triple: lenalidomide, bortezomib, and pomalidomide or lenalidomide, bortezomib, and carfilzomib), or four (quadruple: lenalidomide, bortezomib, pomalidomide, and carfilzomib) previous lines of treatment were ineffective, defined as documented disease progression during or within 60 days of completing their last anti-myeloma therapy.

^¶^In the VenV arm, 47% of patients received 1 prior line of therapy and 53% received 2–3 prior lines of therapy.

Abbreviations: ASCT, autologous stem cell transplant; Cy, cyclophosphamide; D, daratumumab; d, dexamethasone; Dox, doxorubicin; E, elotuzumab; Isa, isatuximab; IMiD, immunomodulatory drug; K, carfilzomib; N, ixazomib; NMA, network meta-analyses, NR, not reported; P, pomalidomide; Pan, panobinostate; Pembro, pembrolizumab; PEG-Dox, pegylated liposomal doxorubicin; PI, proteasome inhibitor; R, lenalidomide; S, selinexor; SAE, serious adverse event; V, bortezomib; Ven, venetoclax; Vor, vorinostat

Lenalidomide and bortezomib are routinely used in clinical practice for MM treatment, including in combination. Assuming all control backbone therapies are “equivalent” when the efficacy differs substantially between studies invalidates this approach, as shown for example by the PFS of CASTOR (Vd) vs POLLUX (Rd). It fails to accurately capture their respective benefit and the role they may play in the efficacy of the regimen. This is also relevant for ICARIA, CANDOR, and KEYNOTE-183, which included neither lenalidomide nor bortezomib in the treatment regimens, but still had their backbone therapies combined for inclusion in the NMA.

This equivalency assumption is likely to also skew the interventional treatment’s adverse event (AE) profile, which may be over- or underestimated based on the backbone regimen. Further, results of the toxicity comparison are rendered misleading by using grade III/IV events interchangeably with SAEs. These two terminologies have distinct definitions and capture different aspects of treatment toxicity. AEs are graded I–V, and seriousness of the event is determined independently of the grade. Generally, the occurrence of grade III/IV AEs is higher than SAEs.

In conclusion, we commend the authors in attempting to address an important open question in the treatment of relapsed myeloma patients; however, we recommend an updated analysis be conducted taking into account the points mentioned above, and validated with clinical experts and experts in evidence synthesis.
